# MiR-34a Interacts with Cytochrome c and Shapes Stroke Outcomes

**DOI:** 10.1038/s41598-020-59997-y

**Published:** 2020-02-24

**Authors:** Heng Hu, Emily A. Hone, Edward A. P. Provencher, Samuel A. Sprowls, Imran Farooqi, Deborah R. Corbin, Saumyendra N. Sarkar, John M. Hollander, Paul R. Lockman, James W. Simpkins, Xuefang Ren

**Affiliations:** 1 Departments of Physiology and Pharmacology, Center for Basic and Translational Stroke Research; West Virginia University, Morgantown, West Virginia 26506 USA; 2 Neuroscience, Center for Basic and Translational Stroke Research; West Virginia University, Morgantown, West Virginia 26506 USA; 3 Microbiology, Immunology and Cell Biology, Center for Basic and Translational Stroke Research; West Virginia University, Morgantown, West Virginia 26506 USA; 4 Human Performance, School of Medicine, Center for Basic and Translational Stroke Research; West Virginia University, Morgantown, West Virginia 26506 USA; 5 Department of Basic Pharmaceutic Sciences, School of Pharmacy, Center for Basic and Translational Stroke Research; West Virginia University, Morgantown, West Virginia 26506 USA; 6 Experimental Stroke Core, Center for Basic and Translational Stroke Research; West Virginia University, Morgantown, West Virginia 26506 USA

**Keywords:** Neuroscience, Blood-brain barrier, Cell death in the nervous system, Cellular neuroscience, Molecular neuroscience

## Abstract

Blood-brain barrier (BBB) dysfunction occurs in cerebrovascular diseases and neurodegenerative disorders such as stroke. Opening of the BBB during a stroke has a negative impact on acute outcomes. We have recently demonstrated that miR-34a regulates the BBB by targeting cytochrome c (CYC) *in vitro*. To investigate the role of miR-34a in a stroke, we purified primary cerebrovascular endothelial cells (pCECs) from mouse brains following 1 h transient middle cerebral artery occlusion (tMCAO) and measured real-time PCR to detect miR-34a levels. We demonstrate that the miR-34a levels are elevated in pCECs from tMCAO mice at the time point of BBB opening following 1 h tMCAO and reperfusion. Interestingly, knockout of miR-34a significantly reduces BBB permeability, alleviates disruption of tight junctions, and improves stroke outcomes compared to wild-type (WT) controls. CYC is decreased in the ischemic hemispheres and pCECs from WT but not in miR-34a^−/−^ mice following stroke reperfusion. We further confirmed CYC is a target of miR-34a by a dural luciferase reporter gene assay *in vitro*. Our study provides the first description of miR-34a affecting stroke outcomes and may lead to discovery of new mechanisms and treatments for cerebrovascular and neurodegenerative diseases such as stroke.

## Introduction

The blood-brain barrier (BBB) is a complex vascular interface that maintains cerebral homeostasis by keeping blood and blood solutes out of the central nervous system (CNS)^1^. This barrier is maintained by cerebrovascular endothelial cells (CECs) that express a high degree of tight junctional protein complexes, which effectively seal off the paracellular route for solute penetration into brain. Dysfunction of the BBB is observed in cerebrovascular diseases and neurodegenerative disorders including stroke, epilepsy, Alzheimer’s, Parkinson’s and Huntington’s diseases^2^. We have recently discovered that maintenance of mitochondrial energy production is critical to maintaining BBB integrity in an *in vitro* CEC culture model, an *in vivo* epidural application model, and an *in vivo* experimental stroke model^3,4^. Another group has also demonstrated a mitochondrial mechanism that regulates BBB integrity and permeability using oxygen-glucose deprivation and re-oxygenation in an *in vitro* model of ischemic reperfusion injury^5^.

Stroke is a devastating neurodegenerative disease that has limited treatment options, and often results in lifetime disability. Primary ischemic stroke damage is caused by the occlusion of the blood vessels, which leads to an insufficient energy supply to the CNS causing neuronal death and neurological damage^6^. Secondary stroke damage is due to the well-documented post-stroke BBB damage, which causes vasogenic edema, disrupts the CNS entry route of energy sources, and is associated with ischemic exacerbation. Thus, regulation of BBB damage may control stroke pathology and lead to therapeutic interventions for stroke.

MiR-34a an important microRNA involved in many pathological and physiological processes such as neural morphology^7^, neurite outgrowth and synapse formation^8^, p53 tumor suppressor network^9,10^, *etc*. We have recently discovered that miR-34a regulates BBB permeability and mitochondrial function using *in vitro* models^11^. In the present study, we employed a strain of miR-34a knockout mice and investigated the role of miR-34a in affecting stroke outcomes using a transient middle cerebral artery occlusion (tMCAO) model. Herein we provide the first evidence that miR-34a is significantly increased in purified pCECs from ischemic hemispheres by tMCAO at 6 h post-stroke reperfusion, which is consistent with the initial BBB opening time point in a stroke. We show deficiency of miR-34a alleviates BBB damage, reduces stroke infarction, and protects from neurological deficits. In addition, we evaluated CYC expression in pCECs from the hemispheres of WT and miR-34a^−/−^ mice and detected a multiple-fold decrease of CYC in pCECs isolated from ischemic WT mice. These data provide evidence for a previously unknown miR-34a-mediated mechanism in a stroke, and may open an entirely new therapeutic strategy for the treatment of acute stroke.

## Results

### miR-34a is involved in ischemia-induced BBB opening

**Figure 1:**
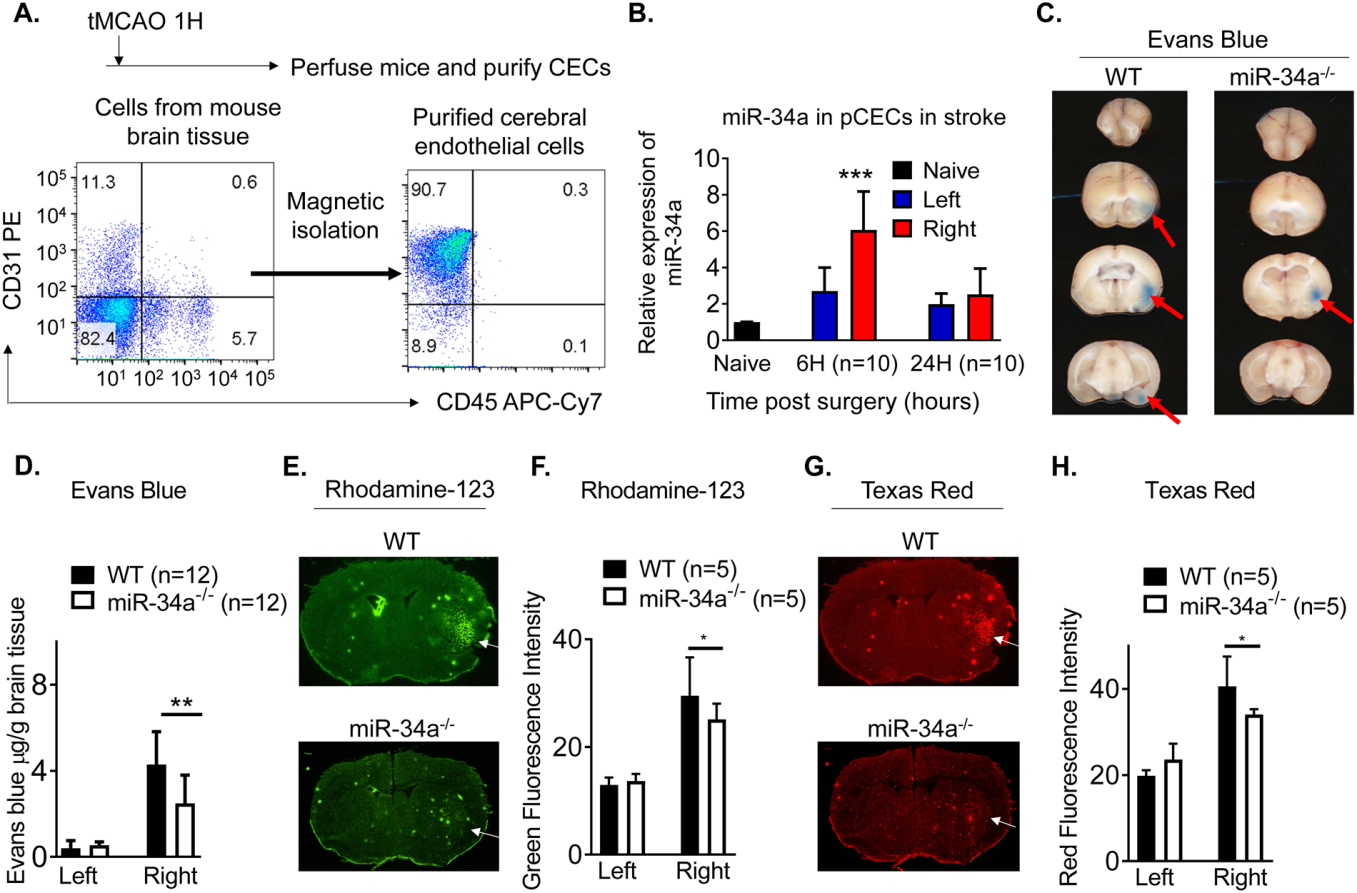
miR-34a is involved in transient BBB opening following murine experimental stroke. (**A**) Flow cytometry data showed purity of magnetically isolated pCECs used to collect the data in (**B**), which showed a significant increase in miR-34a at 6 h post-stroke and no significant change at 24 h post-stroke following 1 h tMCAO. Expression of miR-34a was normalized to internal control miR-39. The data are normalized to naive controls. N = 10 per time point, ***p < 0.001, and one-way ANOVA followed by post hoc Tukey’s test was used for data analysis. Data are expressed as mean ± S.D. (**C**) The middle cerebral artery was occluded for 1 h in miR34a^−/−^ mice and WT controls. Evan’s blue administered intravenously at 6 h post-stroke. Transcardial perfusion was performed, and brain images were photographed as shown by the representative coronal brain sections. Red arrows indicate Evan’s blue extravasation. (**D**) Quantified Evan’s blue extravasation in the left and right hemispheres showed miR-34a depletion decreased BBB permeability, as quantified by µg/g brain tissue in each hemisphere. **p < 0.01, n = 12 per group, and one-way ANOVA followed by post hoc Tukey’s test was used for data analysis. Representative coronal brain images showing BBB opening detected by rhodamine-123 infiltration) (**E**) and Texas Red infiltration (**G**) ischemic brains from WT and miR-34a^−/−^ mice following 1 h tMCAO and 6 h reperfusion. White arrows indicate fluorescent dye infiltration. Quantification of green fluorescence intensity for rhodamine-123 infiltration (**F**) and red fluorescence intensity for Texas Red infiltration (**H**). *p < 0.05, n = 5 per group, and one-way ANOVA followed by post hoc Tukey’s test was used for data analyses.

Previously, we have demonstrated that the BBB is opened at 6 h but closed at 24 h post-tMCAO^12^. We have determined that miR-34a was significantly elevated in the serum of tMCAO mice at 6 h (BBB opening time point) and decreased at 24 h (BBB closing time point)^13,14^. We have also observed that miR-34a levels were significantly elevated in brain ischemic hemispheres at 6 h and 24 h post-stroke reperfusion^14^, but not significantly changed at 48 h and 72 h post-stroke reperfusion (data not shown). To investigate whether miR-34a is involved in BBB opening in early stroke, we purified pCECs from hemispheres of stroke mice (Fig. 1A), and assessed miR-34a levels from pCECs. We found that elevated miR-34a was most prominent in pCECs from ipsilateral hemispheres at 6 h post-tMCAO (Fig. 1B), consistent with the BBB opening time point. The data suggest the potentially important regulatory effects of miR-34a in BBB disruption during the early phase of stroke reperfusion.

To further investigate the role of miR-34a in BBB opening following a stroke, we employed miR-34a knockout mice. Deficiency of miR-34a was confirmed by miR-34a expression in blood by real-time PCR (Supplemental Fig. 1). We confirmed that the vessel anatomy does not differ in wild-type (WT) mice and miR-34a^−/−^ mice by analyzing the distance of the anastomotic points between the anterior cerebral artery (ACA, at 4 mm) and the middle cerebral artery (MCA, at 6 mm) from the midline (Supplemental Fig. 2A,B). We also determined cerebral blood flow changes using a Laser Speckle Imager, and observed no significant difference between the WT and miR-34a^−/−^ mice pre-stroke, post-stroke, and after reperfusion (Supplemental Fig. 2C,D). The physiological parameters, including arterial blood pH value, PaCO2 and PaO2, mean artery blood pressure, body temperature, and blood glucose did not significantly differ between genotypes at pre-stroke, post-stroke, and after reperfusion (Supplemental Table 1). This information indicates miR-34a^−/−^ mice are a suitable model for the stroke study.

To investigate that miR-34a is affecting BBB permeability in a stroke, we performed Evan’s blue extravasation assay on miR-34a^−/−^ mice compared to WT controls following 1 h tMCAO and 6 h reperfusion. We found that BBB permeability was significantly decreased in right hemispheres of miR-34a^−/−^ mice, where the occlusion was localized **(**Fig. 1C,D**)**. We further investigated the BBB permeability by Rhodamine and Texas red detection at 6 h post-tMCAO. Consistently with the Evan’s blue extravasation assay, the data showed that miR-34a^−/−^ mice had significantly less Texas Red (Fig. 1E,F) and rodamine-123 (Fig. 1G,H) infiltration in the brains than WT mice following 1 h tMCAO and 6 h reperfusion (Fig. 1E).

**Figure 2:**
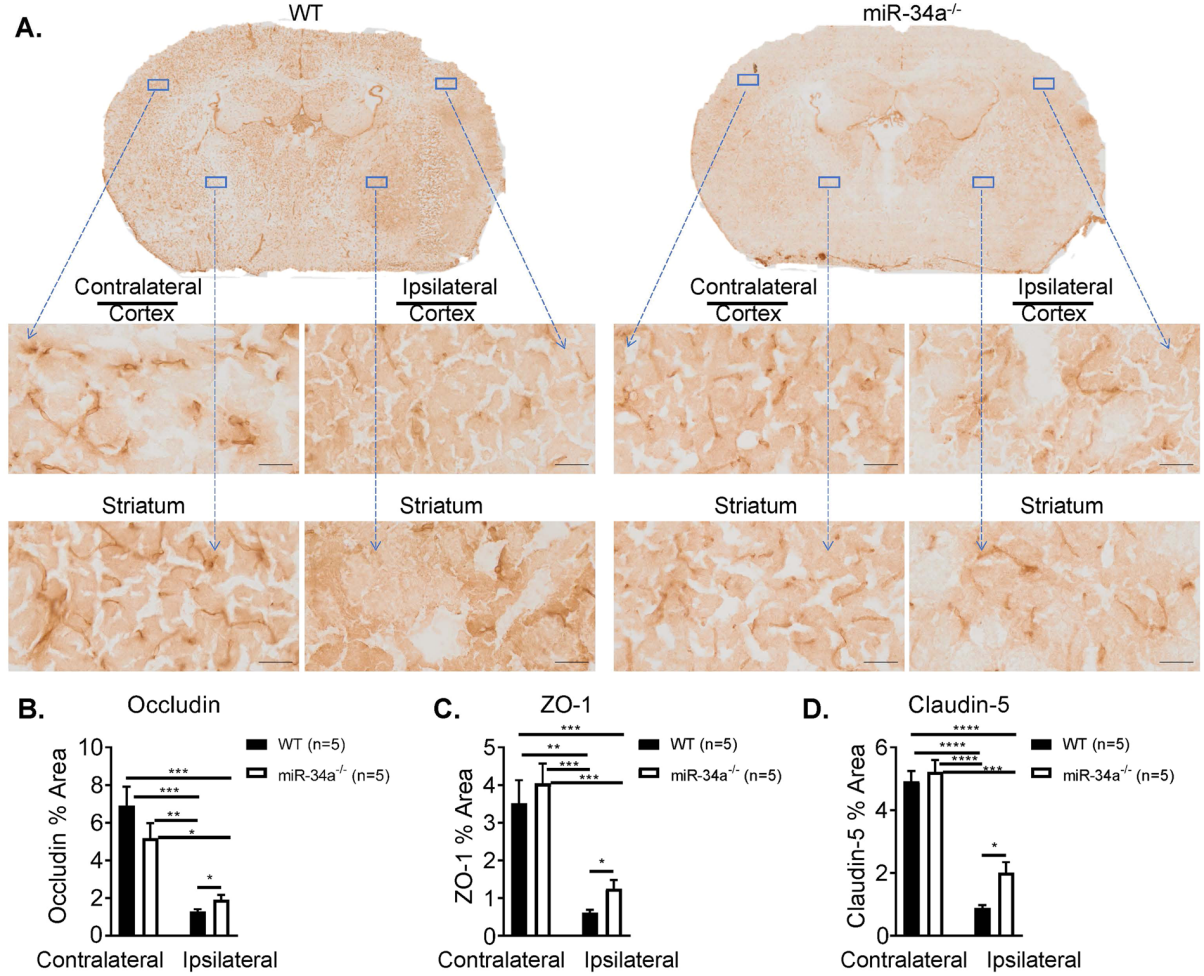
miR-34a depletion alleviates tight junction disruption following murine experimental stroke. Immunohistochemical staining of coronal cryosections of ischemic brains from WT and miR-34a^−/−^ mice following 1 h tMCAO and 6 h reperfusion. (**A**) Representative images for Occludin immunoreactivity detected in both contralateral and ipsilateral hemispheres including cortex and striatum area. Scale bar = 100 µm. Quantification of Occludin (**B**), ZO-1 (**C**), and Claudin-5 (**D**). ****p < 0.0001, ***p < 0.001, **p < 0.01, *p < 0.05, n = 5 per group, and one-way ANOVA followed by post hoc Tukey’s test was used for data analyses.

As tight junctions are dynamic structures that contribute the BBB and affect BBB permeability^15^, we performed immunohistochemistry staining for tight junction proteins, including Occludin, Zonula occludens-1 (ZO-1), and Claudin-5 in ischemic brains of miR-34a^−/−^ vs. WT mice following 1 h tMCAO and 6 h reperfusion **(**Fig. 2). The quantified data suggested overwhelmed loss of tight junctions in ischemic hemispheres compared to contralateral hemispheres. Interestingly, deficiency of miR-34a caused less extent of loss tight junctions than WT mice shown by Occludin (Fig. 2B), ZO-1(Fig. 2C), and Claudin-5 staining (Fig. 2D). These data suggest knockout of miR-34a protects BBB integrity and alleviates BBB permeability following ischemic stroke reperfusion.

### Deficiency of miR-34a alleviates stroke infarction and neurological deficits

**Figure 3:**
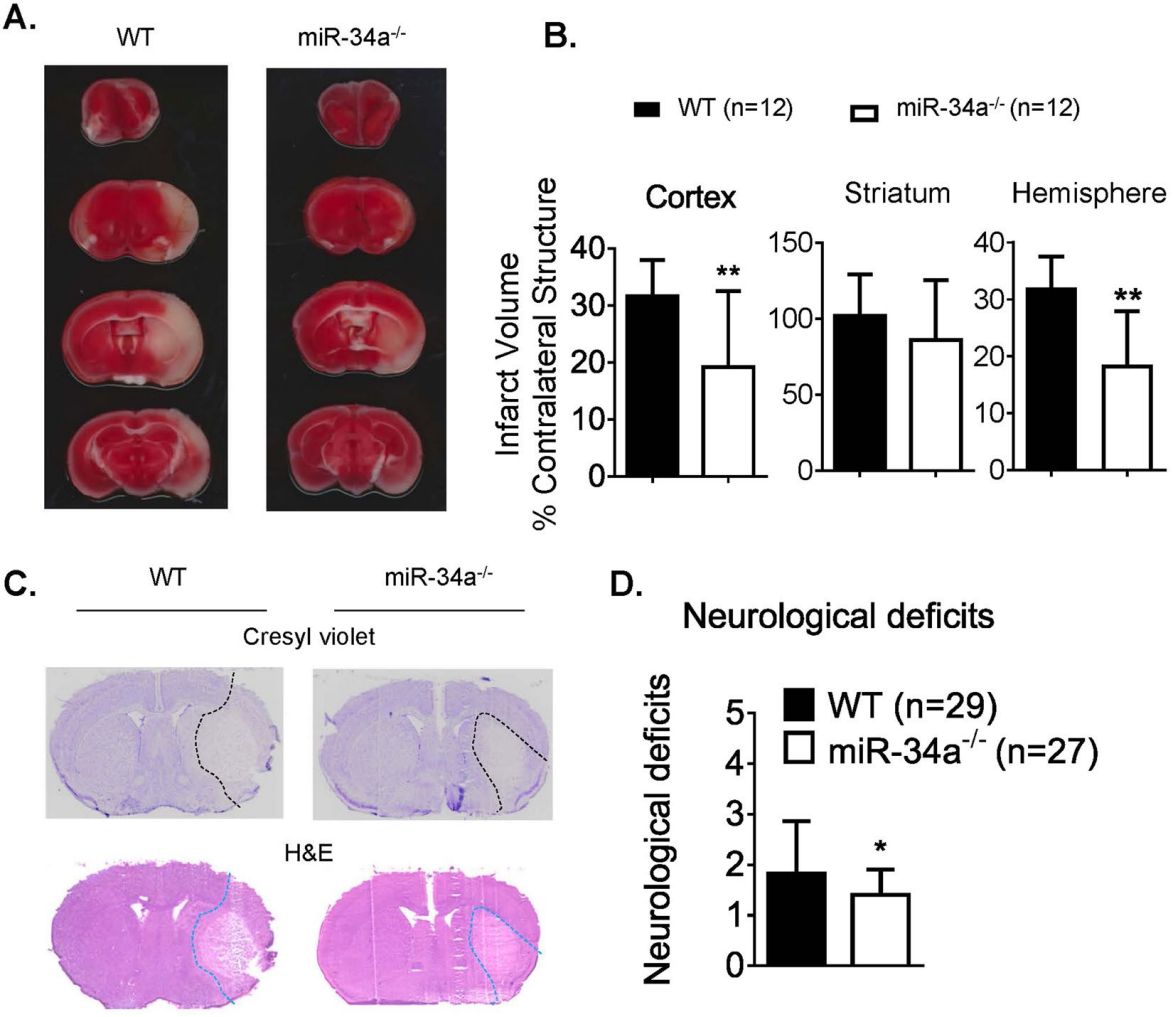
miR-34a depletion reduces stroke infarction and improves neurological deficits. (**A**) Representative TTC staining from WT and miR-34a^−/−^ mice following 1 h tMCAO and 24 h reperfusion. (**B**) Quantified infarct volumes showed a significant decrease in miR-34a^−/−^ mice, **p < 0.01 WT vs miR-34a^−/−^ mice (n = 12 per group). Student’s t test (two-tailed) was used for data analysis. (**C**) Representative Cresyl violet staining (infarction outlined by black dash lines) and H&E staining (infarction outlined by blue dash lines) from coronal brain sections of WT and miR-34a^−/−^ mice following 1 h tMCAO and 24 h reperfusion. (**D**) Neurological deficits of animals generated data for stroke infarction and BBB permeability at 6 h and 24 h end-points. *p < 0.05, WT (n = 29) vs miR-34a^−/−^ (n = 27) mice. Student’s t test (one-tailed) was used for data analysis. Data are expressed as mean ± S.D.

To test the role of miR-34a affecting stroke outcomes, we subjected a cohort of miR-34a^−/−^ and WT mice to 1 h tMCAO and analyzed infarct volume. We observed a significant decrease in the cortex and total hemisphere from miR-34a^−/−^ mice compared to WT controls **(**Fig. 3A,B**)**. The stroke infarction is confirmed by Cresyl violet staining and H&E staining (Fig. 3C). We also demonstrated that miR-34a^−/−^ mice showed better neurological function compared to WT controls at both 6 h and 24 h end-points **(**Fig. 3D**)**. These data clearly demonstrated deficiency of miR-34a improves stroke outcomes.

### miR-34a interacts with cytochrome c in stroke

**Figure 4:**
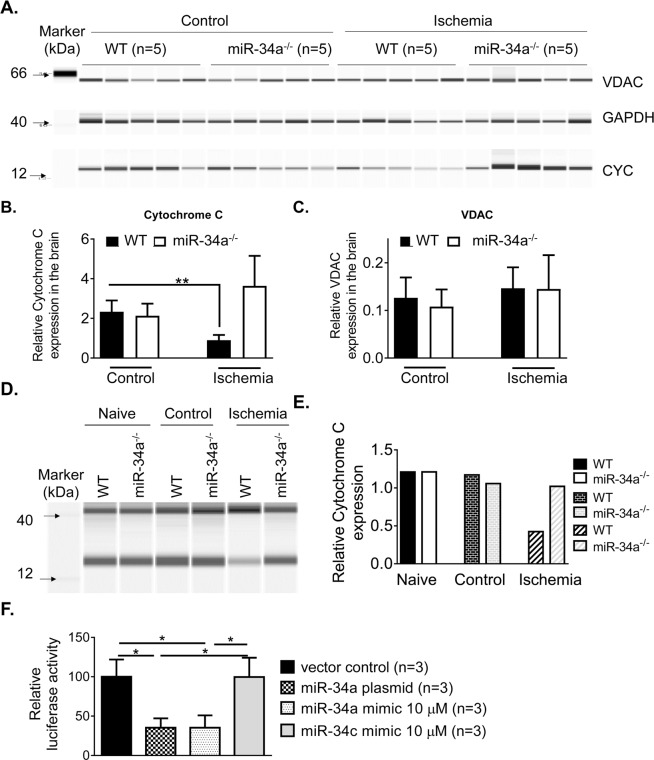
MiR-34a affects stroke outcomes via interacting with cytochrome c. (**A**) WES system image showing CYC, VDAC and GAPDH expression from hemispheres of WT and miR-34a^−/−^ mice at 6 h post-stroke. **(B)** Relative CYC expression normalized to GAPDH from the data generated by the WES system. CYC level is significantly decreased in ischemic hemispheres of WT mice but no significant changes in miR-34a^−/−^ mice between contralateral hemispheres (Control) and ischemic hemispheres (Ischemia). N = 5 per group, **p < 0.01, One-way ANOVA followed by post hoc Tuckey’s test was used for data analysis. Data are expressed as mean ± S.D. **(C)** Relative VDAC expression normalized to GAPDH from the data generated by the WES system. VDAC was not significantly altered in WT mice nor miR-34a^−/−^ mice. (**D**) Multiplexed WES system image showing CYC and GAPDH expression from purified pCECs of WT and miR-34a^−/−^ mice (n = 10 per group, pooled cell samples) at 6 h post-stroke. (**E**) Relative CYC expression by normalization to GAPDH. A 2.8 fold decrease of CYC level was observed in WT mice but no changes were observed in miR-34a^−/−^ mice between contralateral hemispheres and ischemic hemispheres. (**F**) A CYC reporter was coexpressed with a miR-34a plasmid, a miR-34a mimic, a miR-34c mimic, or a plasmid control in cultured cerebral vascular endothelial cells for 24 hours. Relative firefly luciferase activity was evaluated and normalized to renilla luciferase activity. Relative firefly luciferase activity was reduced by miR-34a plasmid and miR-34a mimic. The experiment was repeated 3 times and triplicates were used for each analysis. Data represents the mean ± S.D. *p < 0.05. One-way ANOVA followed by post-hoc Tuckey’s test was used for analysis.

*In vitro*, miR-34a transfection was shown to trigger a reduction of mitochondrial oxidative phosphorylation (OxPhos) and ATP production, along with decreased CYC levels^11^. To investigate whether miR-34a affects stroke outcomes by interacting with CYC *in vivo*, we subjected WT and miR-34a^−/−^ mice to 1 h tMCAO following 6 h reperfusion, and measured CYC levels from brain samples using a single-plexed WES system. We observed that there is a significant decrease of relative CYC expression in ipsilateral hemispheres as compared to contralateral hemispheres in WT mice but not in miR-34a^−/−^ mice **(**Fig. 4A,B). However, the off-target control mitochondrial candidate, voltage-dependent anion channel (VDAC) was not significantly altered (Fig. 4A,C). To further address the specific role of miR-34a in this mechanism, we purified pCECs using magnetic beads and designed a multiplexed WES system to assess the relative expression of CYC from miR-34a^−/−^ mice and WT controls. Interestingly, an approximate 2.8 fold decrease of CYC level was observed from pCECs of WT mice but no changes in miR-34a^−/−^ mice between contralateral hemispheres and ischemic hemispheres **(**Fig. 4D,E**)**.

To validate CYC as a target of miR-34a, pmirGlo vector (Supplementary Fig. 3) was employed and CYC gene (Supplementary Fig. 4 containing miR-34a targeted bases) was constructed for pmirGlo-CYC reporter for a dural luciferase reporter gene assay. The pmirGlo-CYC reporter was coexpressed with miR-34a plasmid, miR-34a mimic, miR-34c mimic, or vector control in cultured cerebral vascular endothelial cells for 24 hours. The data demonstrated that miR-34a mimic or miR-34a plasmid reduced relative firefly luciferase activity in cultured cerebral vascular endothelial cells compared to controls at 24 h post-transfection (Fig. 4F), however the miR-34c mimic did not significantly alter relative firefly luciferase activity compared to controls. Interestingly, several groups reported a decrease of CYC in the mitochondrial fraction, and its translocation to the cytosol from stroke brain tissue analyzed by traditional Western-blot^16–18^. These data suggest the possibility that miR-34a is involved in the decrease of CYC at the translational level.

## Discussion

In this study, we demonstrate a very interesting and novel finding that miR-34a triggers the breakdown of the BBB in murine experimental stroke, paralleled by increased miR-34a level in pCECs from stroke mice. Furthermore, we elucidate that miR-34a affects stroke outcomes and interacts with CYC following stroke reperfusion using miR-34a knockout mice. The results revealed a novel mechanism of miR-34a affecting stroke outcomes.

It has been documented that miRNAs may regulate BBB permeability^11^ and affect mitochondrial structure and function^19^. miR-155^20^, miR-181c^21^, and miR-29c^22^ negatively affect BBB openings. miR-181c regulates mitochondrial morphology^23^; miR-1 affects mitochondrial mRNA translation^24^; miR-181 regulates multiple Bcl-2 family members and mitochondrial function^25^; and miR-29a targets VDAC and affects mitochondrial function^26^. All of these are involved in OxPhos energy metabolism. Our recent work demonstrates OxPhos in CECs plays a critical role in opening of the BBB following stroke^3^. Therefore, regulating OxPhos in CECs may affect BBB permeability and stroke outcomes. MiR-34a plays such a role in reducing OxPhos and increasing BBB permeability in cultured CECs^11^. Our data demonstrate the increase of miR-34a expression in pCECs at the time point of opening of the BBB following stroke reperfusion (Fig. 1). Furthermore, we demonstrate knockout of miR-34a reduces BBB permeability (Fig. 1**)** and protects disruption of BBB tight junctions **(**Fig. 2), suggesting the critical role of miR-34a in regulating BBB opening in stroke *in vivo*. Interestingly, rhodamine-123, a P-glycoprotein-mediated efflux marker is detected in ischemic hemispheres when the BBB is opened (Fig. 1E). However, tissue distribution of rhodamine-123 is not significantly different between normal brain and the metastatic lesion with a blood-tumor barrier opening, which is demonstrated by elevated permeability using a passive diffusion marker ^14^C^27^. As P-glycoproteins are members of ATP-binding cassette transporter superfamily, rhodamine-123 need ATP production to enter through the BBB^28^. The passive diffusion marker, ^14^C does not require ATP production to infiltrate into impaired barriers. These data further suggest that the BBB is opened by impaired OxPhos in CECs after a stroke and miR-34a is involved in the process. The data provide solid and direct evidences that miR-34a plays a critical role in affecting acute stroke outcomes.

It is reported that CYC is released into the cytosol, forms apoptosomes and activates apoptotic pathways in a stroke^29^. Given that CYC transports electrons in respiratory electron transport chain in mitochondria, decrease or loss of CYC in mitochondria may affect electron flow and OxPhos. CYC depletion reduces the capacity of cytochrome c oxidase^30^, a powerful superoxide scavenger within the mitochondrial intermembrane space to function as an antioxidant. Yang *et al*.^31^ has demonstrated that loss of CYC results in mitochondrial dysfunction and impairs the radiation-induced bystander effect. Importantly, Pasdois *et al*.^32^ has also reported that CYC depletion increases production of reactive oxygen species independent of mitochondrial apoptotic pathways such as Bax, Bad, Bak or Bid during heart ischemia. We detected the decrease of CYC in ischemic hemispheres (Fig. 4A,B) and purified pCECs from ischemic hemispheres (Fig. 4D,E) of WT mice. Together with our data, these findings argue that activation of apoptotic pathways by cytosolic CYC may not be the only mechanism of CYC release in stroke exacerbation. While the data suggest a mitochondrial CYC reduction mechanism involved in stroke exacerbation, miR-34a might be a major culprit in decreasing CYC in mitochondria.

To be noted, previous studies^33,34^ show an increase of cytosol CyC and no change in the mitochondria following cerebral ischemia. We used whole-cell lysates from brain tissue and purified endothelial cells (Fig. 4A–E). Even though a different model and traditional Western blot were used in these studies, isolation of cytosol and mitochondrial components may help further clarify the role of miR-34a in regulating CYC expression in cells.

In addition to targeting CYC, our bioinformatic analysis of murine miRNA databases predicts that miR-34a may potentially target and silence other mitochondria-associated mRNAs^11,35^. These gene candidates include Complex I: NADH dehydrogenase ubiquinone 1 subunit C2; Complex II: succinate dehydrogenase complex subunit A and succinate dehydrogenase complex subunit C; Complex III: Ubiquinol-cytochrome c reductase binding protein cytochrome b reductase 1 and Ubiquinol-cytochrome c reductase subunit VII; Complex IV: cytochrome c oxidase 10; pyruvate dehydrogenase kinase isozyme 2, pyruvate dehydrogenase kinase isozyme 2, pyruvate dehydrogenase kinase isozyme 3, lactate dehydrogenase A, and superoxide dismutase 2. Another group recently reported that miR-34a regulates the blood-tumor barrier by targeting protein kinase Cε^36^. The present study did not address and verify other miR-34a targets; however, our novel findings provide generic evidence that miR-34a regulates BBB permeability and changes stroke outcomes, and provide a new explanation for the effects of CYC on stroke pathology. It is important to experimentally verify miRNA’s function as each miRNA may have hundreds of potential targets derived from miRNA databases, but only a small percentage of these targets are effectively suppressed. It is expected that further work and translational evidence will be provided for the field.

One limitation of our current study is that endothelial-specific miR-34a knock out mice are not used in the study. It is well-known that peripheral inflammatory cells play an important role in shaping stroke outcomes. However, our study did not rule out the involvement of inflammatory cells regulated by miR-34a because global knockout of miR-34a might affect peripheral inflammatory responses. We are now generating endothelial specific miR-34a knockout mice to clarify this issue.

The present study describes a previously unknown mechanism of BBB permeability and stroke outcomes. In addition to our basic research, others recently reported a correlation of miR-34a upregulation in clinical stroke patients and experimental stroke rats^37^. Our work suggests that miR-34a is a connection between basic science and translational stroke research that is clinically significant. In summary, our observations suggest a new therapeutic strategy by targeting miR-34a for acute stroke.

## Materials and Methods

### Animals

Homozygous breeders for miR-34a deficient (miR-34a^−/−^ or KO, Stock No. 018279) mice^38^ and C57BL/6 control (WT, Stock No. 000664) mice were obtained from the Jackson Laboratory. The ARRIVE guidelines^39^ were followed to report all animal experiments. To avoid gender difference in response to stroke surgery, we used male mice (3~6 month-old, 25~30 grams) in the experiments. Animals were randomized to experiments. 165 mice were used in the study. Sample size was determined by the analysis of 80% power. All experiments were performed in accordance with National Institutes of Health guidelines and approved by the Animal Care and Use Committee at West Virginia University (WVU), which is accredited by the American Association for Accreditation of Laboratory Care.

### Ischemic stroke model

Animal surgery was performed under isofluorane anesthesia with standard operation procedures from the Experimental Stroke Core at the WVU. The mice were subjected to 1 h transient middle cerebral artery occlusion (tMCAO) using silicon coated MCAO sutures (Cat. #702334, diameter 0.23 mm, Doccol Corporation, MA) followed by reperfusion as published previously^3,4^. Body temperatures were controlled at 37 ± 0.5 °C during occlusion. Occlusion and reperfusion were verified in each animal by a Laser Speckle Imager (Moor Instruments, England). Experimenters were blinded for data analysis. The inclusion/exclusion criteria were followed as we published previously^3,4^. Two WT mice were reported dead before the 24 hour end-point without brain hemorrhage, and the data were included in the neurological deficits. Bupivacaine (2 mg/kg, s.c.) was administered to relieve pain after surgery.

### Isolation of primary cerebrovascular endothelial cells

The procedures of enriching pCECs were adapted from a published protocol^40^. Briefly, animals were perfused transcardially with ice-cold PBS under anesthesia. Brain tissue was collected, washed with HEPES-MEM buffer once, and digested with DNase (Cat. #LK03172, Worthington, NJ) and papain (Cat. #LK003178, Worthington, NJ) for 70 minutes. Then samples were mixed with 30% BSA and centrifuged at 1290 g for 10 min. Myelin was removed from the top of tubes and cells were suspended in 10% FBS-DMEM medium. Cells were then purified with mouse CD31 magnetic beads (Cat. #130-097-419, Miltenyi, Germany) following the manufacturer’s instructions. To verify the cell purity, a portion of the cells were incubated with magnetic beads, APC-Cy7-conjugated anti-mouse CD45 (Cat. #557659, BD Bioscience, CA), and PE-conjugated anti-mouse CD31 (Cat. #553373, BD Bioscience, CA) followed by cell sorting procedures and analyzed by flow cytometry (BD, LSRFortessa).

### Real-time PCR

Total miRNA was isolated from purified primary cerebrovascular endothelial cells (pCECs) using the miRNeasy mini kit protocol (Qiagen, Valencia, CA), and then converted to cDNA using oligo dT, random hexamers with Superscript RT II enzyme (Invitrogen, Grand Island, NY). Real-time PCR was performed using SYBR Green master mix (Qiagen, Valencia, CA) and predesigned primers (synthesized by Qiagen), normalized to internal control miR-39 expression.

### BBB permeability assay *in vivo*

Evan’s blue and fluorescent dye were used as measures for BBB permeability in mice. Evans blue (2% in saline; 4 mL/kg, Sigma, CA), or mixture of Texas Red (6 mg/Kg, Alfa Aesar, MA) and rhodamine-123 (6 mg/Kg, Life Technologies, CA) was intravenously administered through tail vein 30 minutes prior to euthanization. Animals were transcardially perfused with saline and brains were sectioned with a 2 mm brain matrix for Evan’s blue extravasation assay. Hemisphere samples were weighed, homogenized with 400 uL PBS, and precipitated with 50% trichloroacetic acid (Sigma, CA) overnight. All samples were centrifuged at 1000 rpm for 30 minutes to separate out the brain tissue in pellet prior to measuring. Absorption was quantified at 610 nm with a plate reader (BioTek, Winooski, VT). To quantify Evans Blue in the brain, an Evans blue standardized curve was used. Results were quantified as microgram/gram brain tissue. For Rhodamine and Texas red detection, all brains were frozen immediately in −80 °C isopentane. Leica CM30505 Cryostat was used to cut brains slices (20 µm). All brain sections were inspected for fluorescence under a microscope (Olympus MVS10, Japan). Every 5 sections between bregma = 0 mm and bregma = −2 mm were photographed for analysis of mean fluorescence intensity using ImageJ software.

### Immunohistochemical staining and data analyses

Cryosections were pre-cooled with acetone (−20 °C) for 10 min, rinsed with PBS, incubated in 0.3% H_2_O_2_ solution in PBS at room temperature for 10 min, and rinsed with PBS again. Sections were blocked in 10% fetal bovine serum in PBS at room temperature for 1 hour, then incubated with the following primary antibodies: Occludin (1:100, Cat. No 331500, Invitrogen), ZO-1(1:500, Cat. No. 617300, Invitrogen), Claudin-5 (1:100, Cat. No. 352500, Invitrogen) overnight at 4 °C. Slices were rinsed with PBS then stained with HRP Polymer/DAB Plus Chromogen (Cat. No. TL015HD, Thermo Scientific) according to the manufacturer’s instructions. Olympus VS120 Slide Scanner were used to process images under 40X magnification. Every 10 sections between bregma = 0 mm and bregma = −2 mm were used for the analyses of tight junction staining, images were thresholded and the percentage of pixel area of the tight junctions was calculated using ImageJ^41^.

### Quantification of brain infarction

The brain sections were stained with 2% Tetranitroblue tetrazolium chloride (TTC, Cat. #T4000, Sigma, Saint Louis, MO) at 37 °C for 30 minutes then photographed by an image scanner. The infarct volume was analyzed by ImageJ software (National Institutes of Health) in a double-blinded manner. The infarct volume was expressed as a percentage of contralateral cortex, striatum, and total hemisphere. The TTC-stained brain sections were fixed in formalin for one week then embedded in paraffin to obtain brain slices (10 µm). Cresyl Violet and Hematoxylin & Eosin (H&E) staining were performed on paraffin-embedded slices. Bright field images were taken through a slide scanner (Olympus VS120, Japan) at Microscope Imaging Facility at WVU.

### Neurological deficits

Neurological deficits were determined at 6 h and 24 h post-tMCAO according to a 0- to 5-point scale neurological score system as published^42^. All end-point scores were reported. The experimenters were blinded for genotypes.

### Protein analysis by WES system

Protein samples were extracted by 1 × RIPA buffer (#9806 Cell signaling Technology, CA) with 1 mM PMSF (Cat. #8533 Cell Signaling Technology, CA) and normalized to 2 µg/µl. Each sample of 3 µl was assayed using the Simple Western system (WES, Cat. #004–600, Protein Simple, CA). Protein analysis was performed according to the manufacturer’s protocol for the 12–230 kDa separation matrix (Cat. #SM-W004, Protein Simple, CA). Cytochrome c (1:25, Cat. #11940, Cell Signaling Technology, CA), GAPDH (1:500, Novus Biologicals, CO), voltage-dependent anion channel VDAC (1:50, Cat. #4661, Cell Signaling Technology, CA) were used for primary antibody. An anti-Rabbit Detection Module (Cat. #DM-001, Protein Simple, CA) was used according to the manufacturer’s instruction.

### Luciferase reporter gene assay

Cerebral vascular endothelial cells (bEnd.3 cell line originally from ATCC) were grown in 96 well white plate with 5%FCS DMEM medium. pmirGLO-CYC reporter (detailed in Supplementary Information) was co-transferred with miR-34a plasmid^11^, vector control, miR-34a mimic, or miR-34c mimic control using RNAiMAX Reagent (Cat. #13778, Life Technologies, CA) per the manufacturer’s protocol. After transfection, the cells were cultured for 24 hours and a dural-luciferase reporter assay kit (Cat. #E2920, Promeaga, CA) was used for the sequential assay of firefly and Renilla luciferase activity recorded by a plate reader (BioTek, Winooski, VT).

### Statistical analysis

Statistical analysis was performed with Prism 5 software (Graphpad, La Jolla, CA). Differences between groups were analyzed by the unpaired Student’s t test or 1-way ANOVA as indicated in the figure legends.

## Supplemental Information

Supplemental Information.
