# Supplementary material for: MiR-34a Interacts with Cytochrome c and Shapes Stroke Outcomes
Source: Sci Rep. 2020 Feb 24;10:3233. doi: 10.1038/s41598-020-59997-y (PMC7040038; doi:10.1038/s41598-020-59997-y)
Supplement: Supplementary file 1 — Supplemental Information. [file 41598_2020_59997_MOESM1_ESM.pdf]

MiR-34a Interacts with Cytochrome c and Shapes Stroke Outcomes

Heng Hu[1,6], Emily A. Hone[2,3], Edward A.P. Provencher[2], Samuel A. Sprowls[5], Imran Farooqi[2], Deborah R. Corbin[2], Saumyendra N. Sarkar[1], John M. Hollander[4], Paul R. Lockman[5], James W. Simpkins[1,6], Xuefang Ren*[2,3,4]

[1]Departments of Physiology and Pharmacology, [2]Neuroscience, [3]Microbiology, Immunology and Cell Biology, [4]Human Performance, School of Medicine; [5]Department of Basic Pharmaceutic Sciences, School of Pharmacy; [6]Experimental Stroke Core, Center for Basic and Translational Stroke Research; West Virginia University, Morgantown, West Virginia, 26506 USA

***Correspondence to:**
Xuefang Ren, M.D. Ph.D.
Address: Biomedical Research Center Rm.109, 64 Medical Center Dr.
PO Box 9177, Morgantown, WV 26506 USA.
Fax: 1-304-293-7823;
Tel: 1-304-581-1892;
E-mail: xuren@hsc.wvu.edu

Supplementary Materials and Methods

Brain vessel anatomy

A published protocol[1] was followed for the brain vessel anatomy. A mixture of 2ml carbon black ink (1:9 ratio of 46030 and 44314 from Chartpak Inc. MA) was intravenously injected into the mice under deep anesthesia. The animals were euthanized and brains were removed and photographed. Distance of the anastomotic points between the anterior cerebral artery (ACA, at 4mm) and the middle cerebral artery (MCA, at 6mm) from the midline was quantified.

Physiological analysis

Mice were under anesthesia and the femoral artery was cannulated for measurements of arterial blood gases (blood analysis system, Irma Trupoint, Vetlab Supply), blood glucose (Glucometer Microlet, Bayer), blood pressure (pressure monitor BP-1, World Precision Instruments, FL), and cerebral blood flow (LSI, Moor Instruments). Repeated measurements were made throughout the 1 h MCA occlusion period.

pmirGLO-CYC dural-luciferase reporter constructs

pmirGLO-CYC dural-luciferase reporter constructs were created by cloning miR-34a binding sequence into the pmirGLO vector (Cat. # E1330, Promeaga, CA) with firefly luciferase and Renilla luciferase (**Supplemental Fig. 3**). The gene 68 base pairs (ATGAGTAA TTCCACTGCC TTATTTATTA CAAAACAAAT GTCTCATGGC TTTTAATGTA CACCATAATT) were assembled from synthetic oligonucleotides. The plasmid DNA was purified from transformed bacteria and concentration determined by UV spectroscopy. The final construct was verified by sequencing (**Supplemental Fig. 4**). The sequence identity within the insertion sites was 100%.

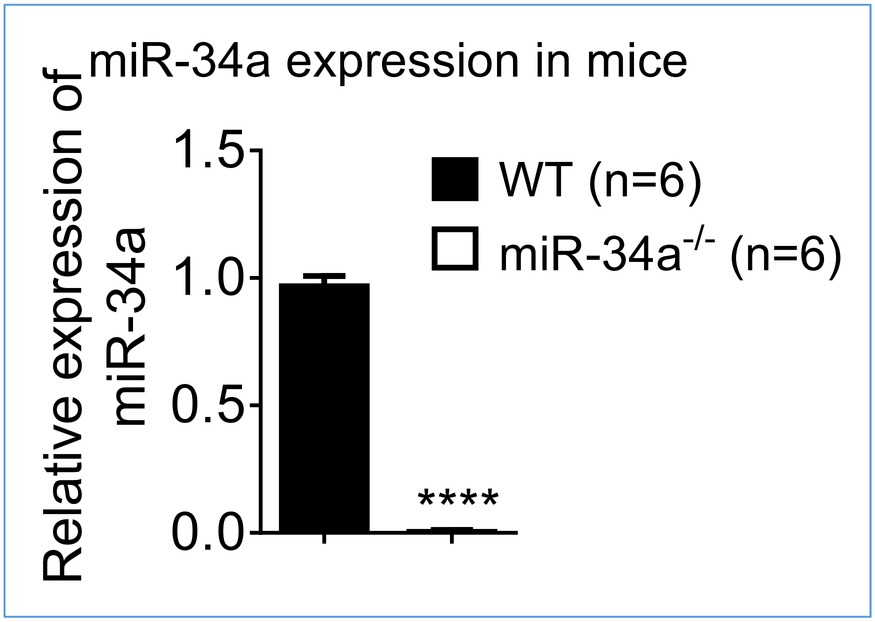

Supplemental Figure 1. miR-34a expression in serum of WT and miR-34a$^{-/-}$ mice. miR-34a expression in serum was quantified by real-time PCR. WT mice showed miR-34a induction in serum, while no miR-34a expression was detected in the serum from miR-34a$^{-/-}$ mice. N=6 per group, ****$p < 0.0001$. Student's t tests are used for data analyses. Data are expressed as mean ± S.D.

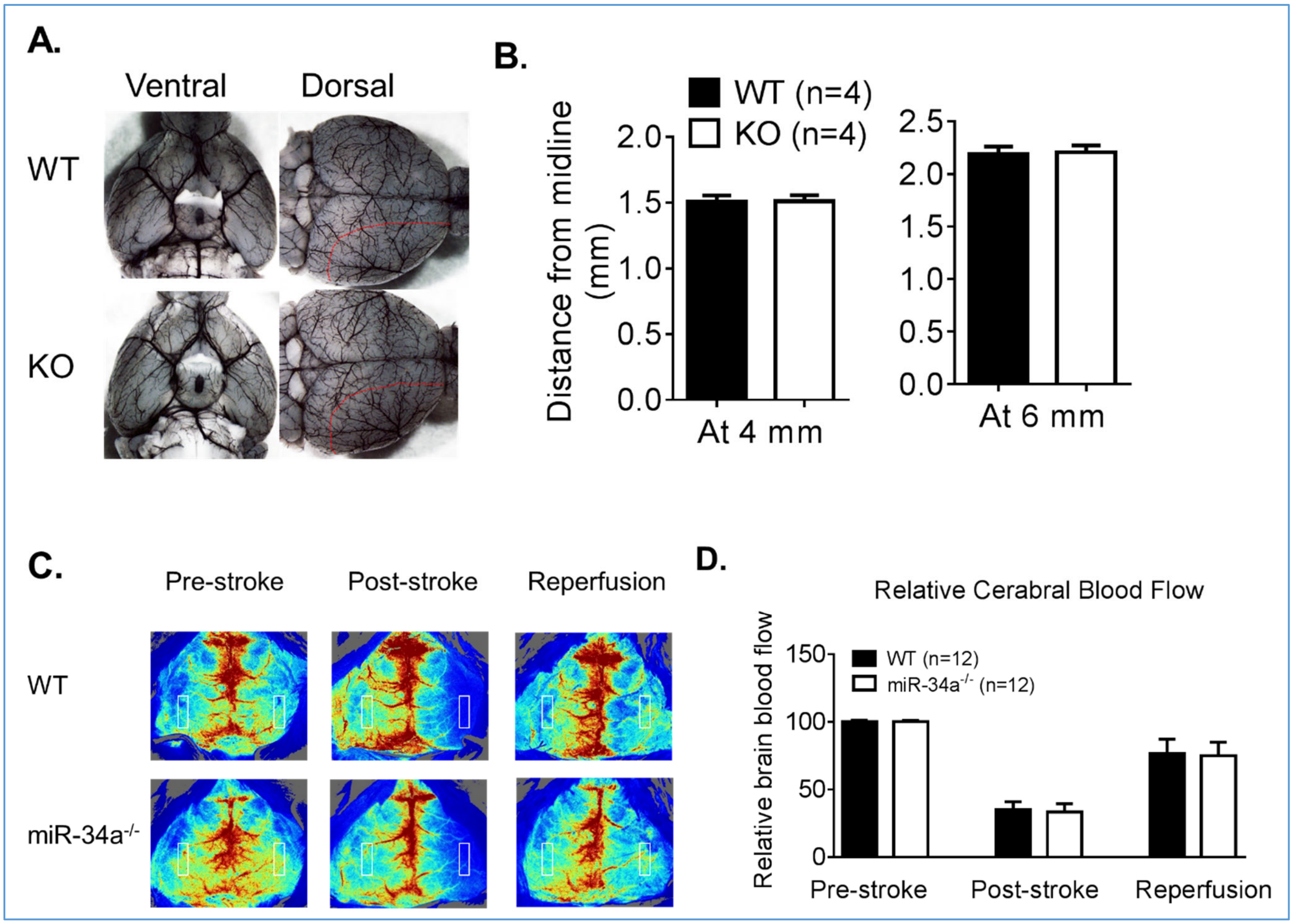

Supplemental Figure 2. miR-34a depletion does not affect cerebral vessel anatomy. (**A**) Whole-brain images showed no significant difference in anatomy, as quantified by (**B**) distance of the anastomotic points between the anterior cerebral artery (ACA, at 4mm) and the middle cerebral artery (MCA, at 6mm) from the midline. (**C**) WT and KO mice were subjected to 1h MCAO, and cerebral blood flow was recorded by Laser Speckle Imager. Representative brain section images showing no difference in cerebral blood flow in WT and miR-34a$^{-/-}$ mice, as quantified by (**D**) relative brain blood flow at pre-stroke, post-stroke, and after reperfusion. Student's t tests are used for data analyses. Data are expressed as mean ± S.D.

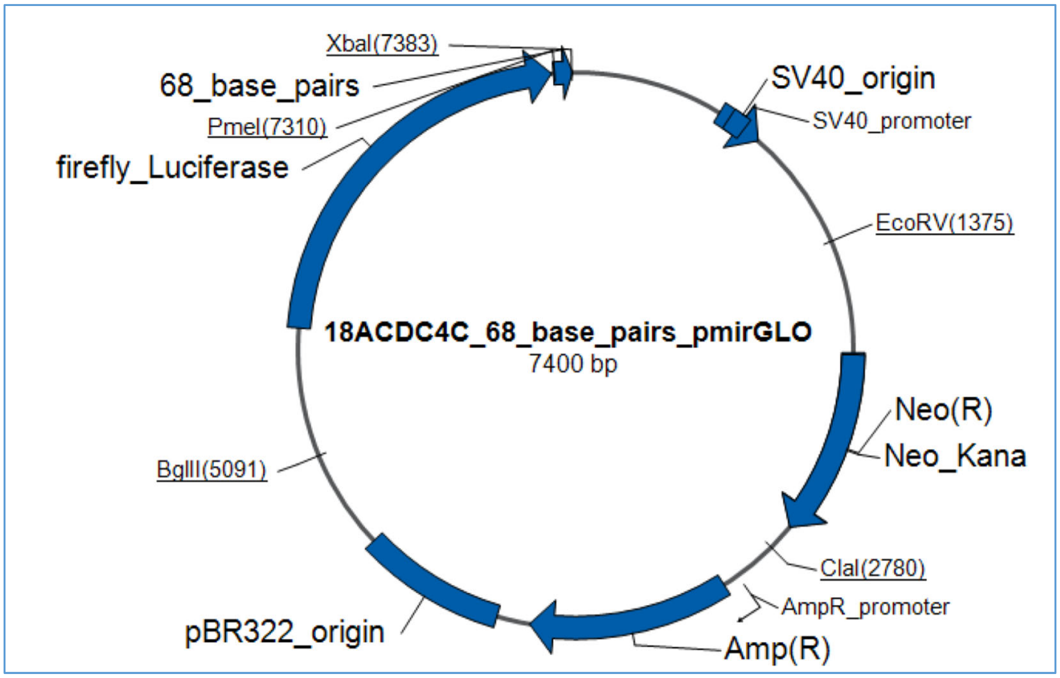

Supplemental Figure 3. The map for pmirGLO-CYC dural-luciferase reporter constructs. The scheme of the synthetic part of CYC gene (68 base pairs) inserted into a dural luciferase reporter vector pmirGLO between PmeI and XbaI sites.

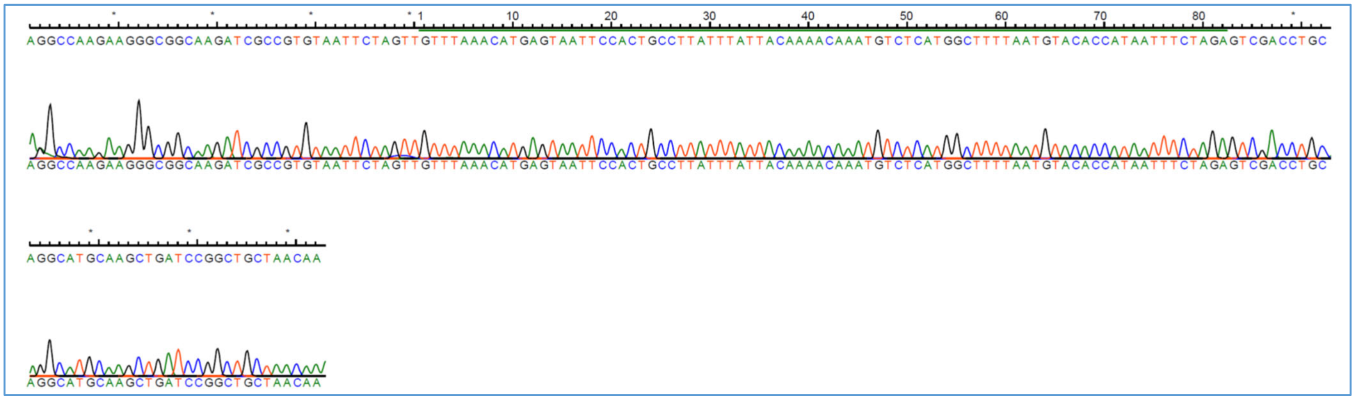

Supplemental Figure 4. The sequence of final construct for CYC luciferase reporter gene. The final construct was verified by sequencing. The sequence identity within the insertion sites was 100%.

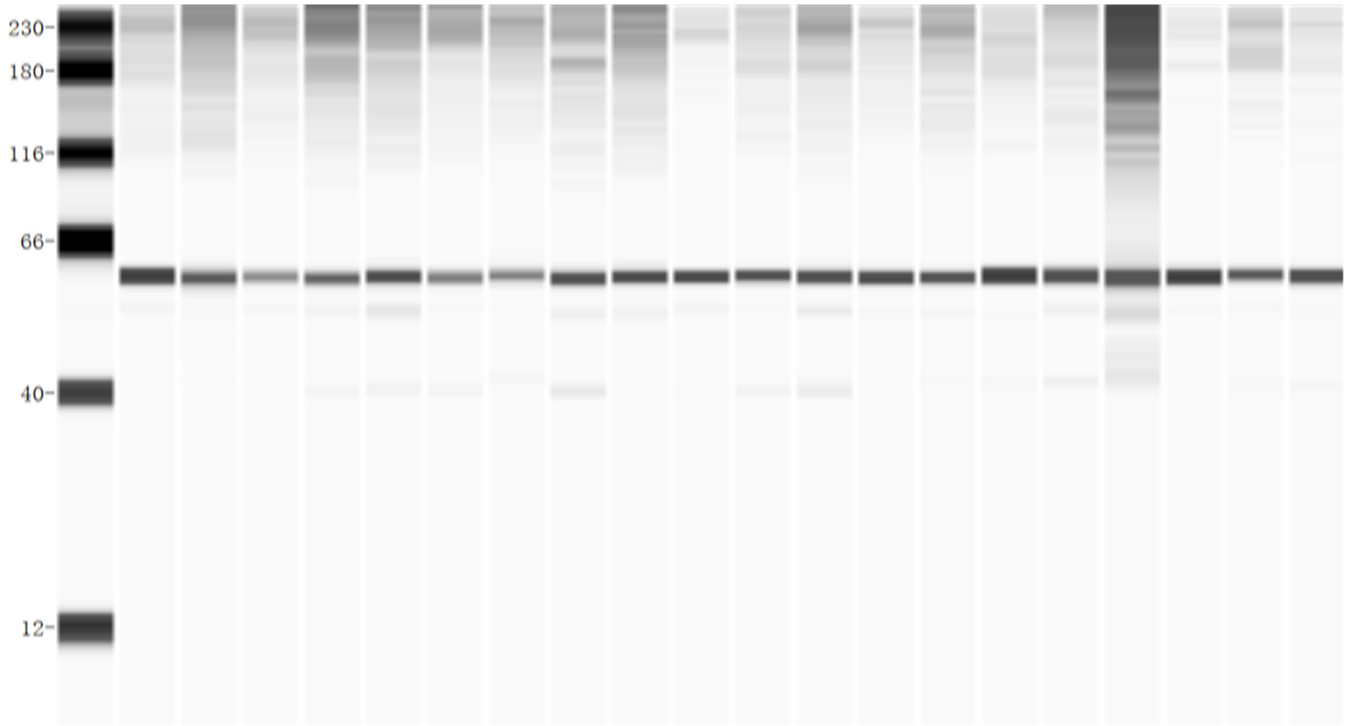

Supplemental Figure 5. The full gel blots for VDAC in Fig. 3A.

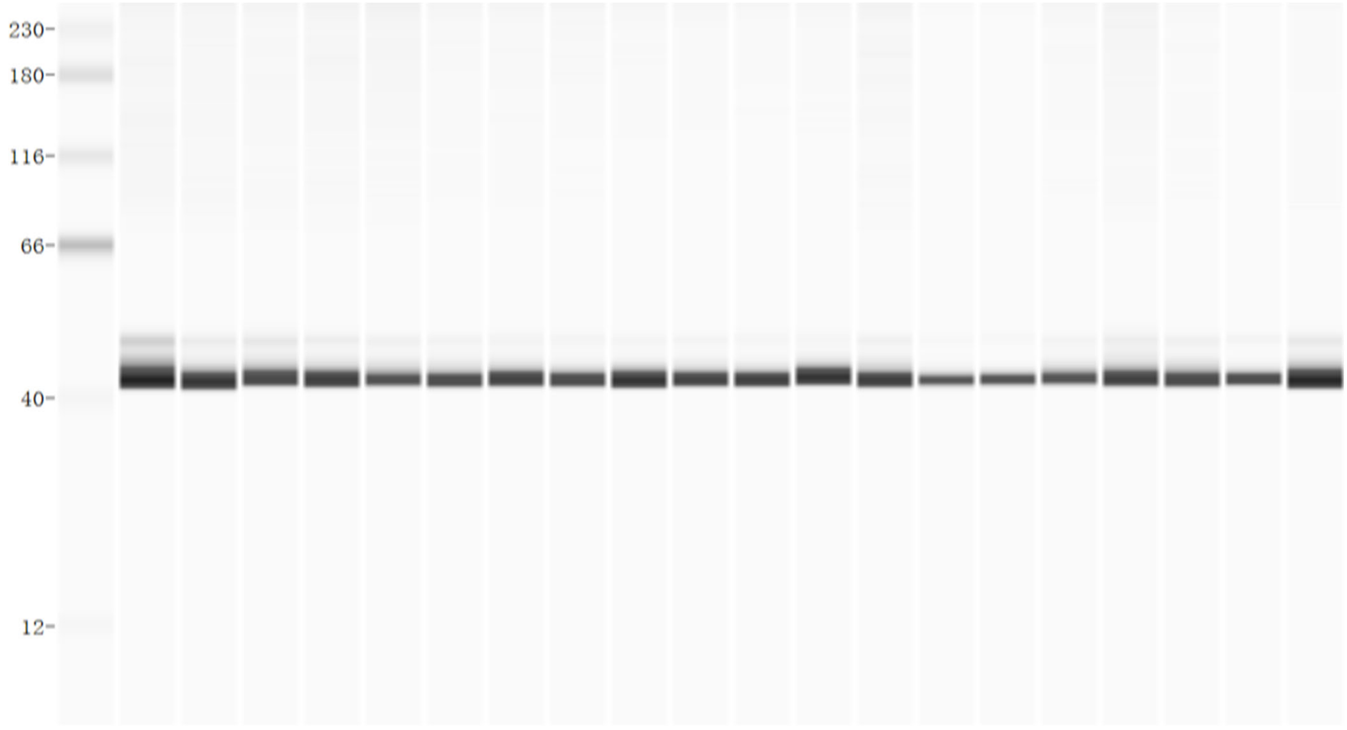

Supplemental Figure 6. The full gel blots for GAPDH in Fig. 3A.

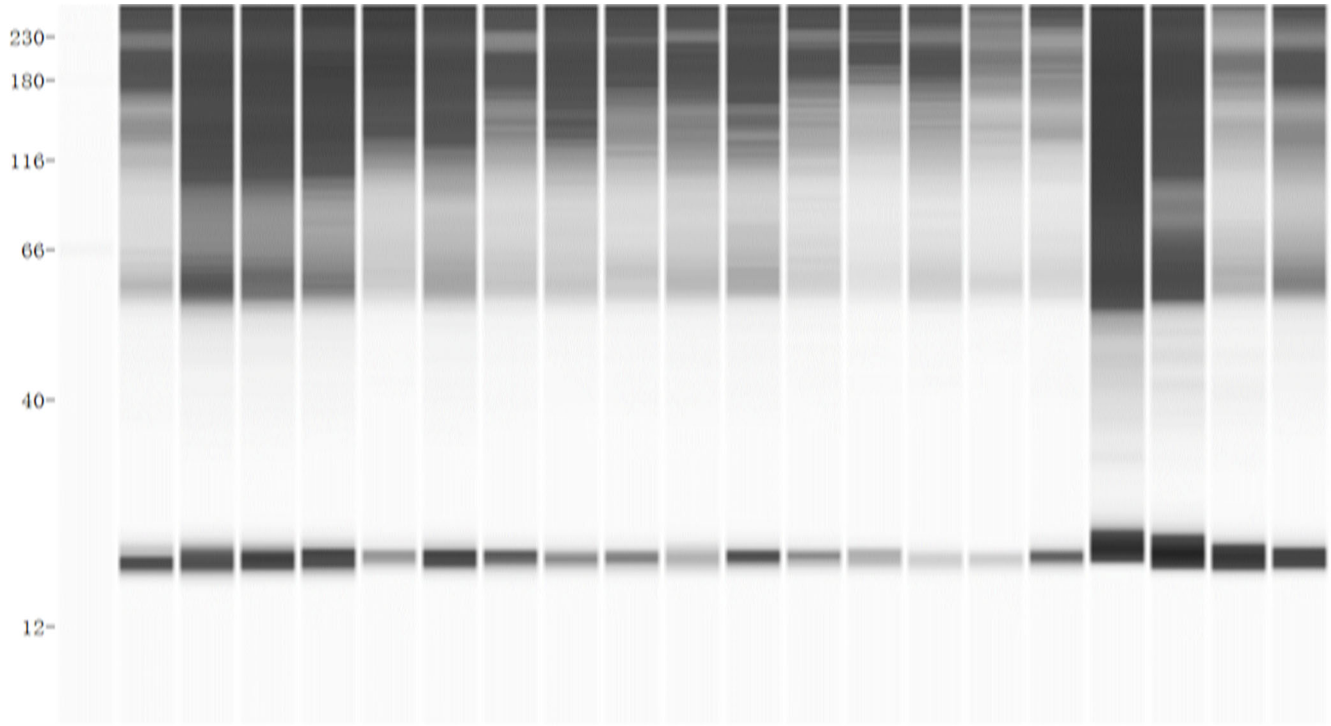

Supplemental Figure 7. The full gel blots for CYC in Fig. 3A.

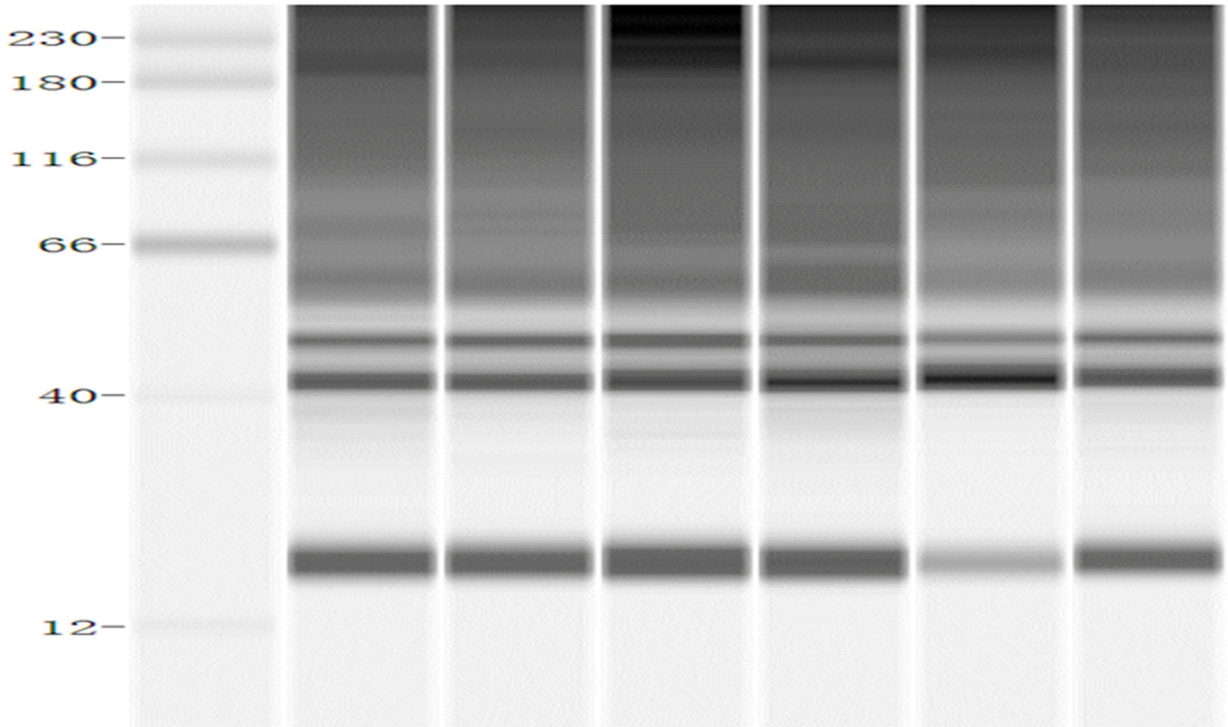

Supplemental Figure 8. The full gel blots for the multiplex blots of GAPDH and CYC in Fig.3D.

Supplemental Table 1. Physiological parameters at pre-, mid- and post-MCAO in WT and miR-34a$^{-/-}$ mice.

Variables	WT (n=4)			miR-34a$^{-/-}$ (n=4)		
	Pre-MCAO	Mid-MCAO	Post-MCAO	Pre-MCAO	Mid-MCAO	Post-MCAO
Arterial blood pH value	7.42 ± 0.05	7.43 ± 0.06	7.38 ± 0.06	7.39 ± 0.04	7.44 ± 0.06	7.40 ± 0.07
Arterial blood CO2 tension (mm Hg)	18 ± 1	24 ± 7	24 ± 5	19 ± 4	24 ± 4	24 ± 8
Arterial blood O2 tension (mm Hg)	174 ± 11	162 ± 23	182 ± 17	177 ± 8	179 ± 17	165 ± 21
Mean artery blood pressure (mm Hg)	84 ± 9	81 ± 4	67 ± 9	83 ± 9	82 ± 4	68 ± 6
Mean artery blood glucose (mg/dL)	112 ± 7	116 ± 9	135 ± 24	109 ± 5	114 ± 11	137 ± 26
Temperature (°C)	37.0 ± 0.4	36.7± 0.3	36.9 ± 0.3	36.7 ± 0.3	36.8 ± 0.3	37.0 ± 0.2

Physiological parameters are not significantly different between the genotypes. Data are presented as mean ± S.D.

Supplemental References

1 Hasan, M. R., Herz, J., Hermann, D. M. & Doeppner, T. R. Intravascular perfusion of carbon black ink allows reliable visualization of cerebral vessels. *Journal of visualized experiments : JoVE*, doi:10.3791/4374 (2013).
